# Equity in healthcare financing: a case of Iran

**DOI:** 10.1186/s12939-019-0963-9

**Published:** 2019-06-17

**Authors:** Faride Sadat Jalali, Abdosaleh Jafari, Mohsen Bayati, Peivand Bastani, Ramin Ravangard

**Affiliations:** 1 Student Research Committee, Shiraz University of Medical Sciences, Shiraz, Iran; 2 Health Management and Economics Research Center, Iran University of Medical Sciences, Tehran, IR Iran; 3 Health Human Resources Research Centre, School of Management and Information Sciences, Shiraz University of Medical Sciences, Shiraz, Iran; 4 Health Human Resources Research Centre, Shiraz University of Medical Sciences, Shiraz, Iran; 5 Health Human Resources Research Centre, School of Management and Medical Information Sciences, Shiraz University of Medical Sciences, Shiraz, Iran; 6 Department of Health Services Management, School of Management and Medical Information Sciences, Shiraz University of Medical Sciences, Shiraz, Iran

**Keywords:** Equity in financing, Health expenses, Gini coefficient, Concentration index, Kakwani index

## Abstract

**Background:**

Fair financial contribution in healthcare financing is one of the main goals and challengeable subjects in the evaluation of world health system functions. This study aimed to investigate the equity in healthcare financing in Shiraz, Iran in 2018.

**Materials and methods:**

This was a cross- sectional survey conducted on the Shiraz, Iran households. A sample of 740 households (2357 persons) was selected from 11 municipal districts using the multi-stage sampling method (stratified sampling method proportional to size, cluster sampling and systematic random sampling methods). The required data were collected using the Persian format of “World Health Survey” questionnaire. The collected data were analyzed using Stata14.0 and Excel 2007. The Gini coefficient and concentration and Kakwani indices were calculated for health insurance premiums (basic and complementary), inpatient and outpatient services costs, out of pocket payments and, totally, health expenses.

**Results:**

The Gini coefficient was obtained based on the studied population incomes equal to 0.297. Also, the results revealed that the concentration index and Kakwani index were, respectively, 0.171 and − 0.125 for basic health insurance premiums, 0.259 and − 0.038 for health insurance complementary premiums, 0.198 and − 0.099 for total health insurance premiums, 0.126 and − 0.170 for outpatient services costs, 0.236 and − 0.061 for inpatient services costs, 0.174 and − 0.123 for out of pocket payments (including the sum of costs related to the inpatient and outpatient services) and 0.185 and − 0.112 for the health expenses (including the sum of out of pocket payments and health insurance premiums).

**Conclusion:**

The results showed that the healthcare financing in Shiraz, Iran was regressive and there was vertical inequity and, accordingly, it is essential to making more efforts in order to implement universal insurance coverage, redistribute incomes in the health sector to support low-income people, strengthening the health insurance schemes, etc.

## Introduction

The World Health Organization (WHO) has declared the fair contribution in healthcare financing as one of the three objectives of the health systems [1]. In case of the lack of a proper financing system, only a limited number of people will have timely access to healthcare services. In fact, the health system financing determines the individuals' ability to access to and buy the health services when they need them [2]. The major sources of health financing in most countries are taxes, social insurance contributions, private health insurance premiums, community financing, and out-of-pocket payments [3]. These methods have different effects on the health system indicators, including equity [4]. In recent years, equity has become one of the top priorities of policymakers and researchers in the world [5]. The International Association for Equity in Healthcare Services defines equity as "the lack of systematic and potentially removable differences in one or more aspects of health in a population and its economic, social and geographical subgroups [6], which can be investigated in three areas of equity in financing, equity in access to services, and equity at the community health level [7]. Equity in health financing can be defined in terms of horizontal and vertical equity, with an emphasis on the vertical equity and in particular the progressivity of financing. An appropriate method for determining the progressivity of financing systems is the use of the indicators that represent payments based on the proportion of income [8]. The Kakwani index, which is in fact derived from the difference between the concentration index (inequality in healthcare payments) and the Gini coefficient (income inequality) is used to understand the progressivity (increases in the health payments with increased income) or regressivity (increases in the health payments with reduced income) of the health financing system [9].

The results of studies in different countries have shown different status in terms of equity in providing financial resources for their health services. For example, the results of studies conducted by Leung et al. (2009) and Crivelli & Salari (2014) showed that the healthcare financing systems in China and Switzerland were generally regressive, and the available vertical inequity had a significant impact on them [10, 11]. However, the results of studies conducted by Mtei et al. (2012) and Yu et al. (2008) showed that the Tanzanian and Malaysian health financing systems were progressive [12, 13]. Abu Zineh et al. in Palestine (2008) reported that the Kakwani index for out-of-pocket payments, private health insurance premiums, and social insurance premiums was, respectively, − 0.08, 0.1 and 0.06 [14].

In Iran, given the requirements of the fifth and sixth development plans for reducing the out-of-pocket payments in the country, the increased contribution of the government in providing the funds for public health expenses and appropriate allocation of public resources in the health sector are of great importance. Fair and equitable provision of healthcare services has been a concern for governments since years ago, but it has always faced serious challenges and obstacles, which have made the goals not be properly achieved. In the eleventh government, great efforts were made to implement it, the result of which was planning, designing and delivering a comprehensive package of the health system transformation in the country. In the perspective of this plan, increasing public satisfaction with health services and reducing out-of-pocket payments have been seen as a top priority [15, 16]. A study by Zarei et al. (2017) reported the rate of out-of-pocket payments by hospitalized patients in public hospitals in accordance with the goal set out in the Health Transformation Plan, indicating the appropriate government support [17]. However, Karami et al. (2018) stated that the large increase in health care tariffs during the implementation of the plan did not lead to a significant reduction in the patients’ out-of-pocket payments, and health insurance mechanisms prevented the plan from reaching its goal of reducing out-of-pocket payments [18].

Few studies have been conducted on the equity in financing the health system in Iran. For example, the results of a study by Oliaeemanesh et al. (2018) showed that financial support in Iran had significantly reduced the income inequality among households, but did not significantly affect equity in the healthcare financing [19]. In the study by Emamgholipour and Agheli (2018), the Kakwani index for urban and rural households has been respectively − 0.572 and − 0.485, implying that the distribution of healthcare costs was regressive and to the detriment of poor households. Therefore, policymakers in the health sector should reduce the overhead costs of low-income households by expanding the health insurance coverage [15]. In their study, Mehrolhassani et al. (2017) concluded that there was no significant legal and policy gap in the Iranian health system financing; however, the implementation methods and commitment to laws had posed fundamental challenges in terms of equity and financial protection [20]. Homayi Rad and Khodaparast (2016) concluded in their study that although the tax system in Iran was progressive, the health insurance mechanism was highly regressive [21]. In the study by Almasian Kia et al. (2015), the Kakwani index for out-of-pocket payments during the study years (2001–2010) was progressive and positive in rural areas, but in urban areas, it was regressive and negative during 2001–2006, and progressive and positive during 2006–2010 [8].

Achieving the goals projected in the health transformation plan requires the adoption of effective corrective policies based on the social realities. Hence, it is important to identify the distribution of medical expenses among the households of the country. To meet this need, the present study was conducted for the first time in 2018 to use the data collected at the households level in Shiraz, Iran in order to investigate and evaluate the equity in health financing in this city, clarify the impacts of related health system reforms such as the Health Transformation Plan over the past years, and provide some suggestions for increasing equity in the health financing.

## Materials and method

### Research design

This was a cross-sectional household survey conducted in Shiraz, Iran in 2018. Shiraz is the fifth-most-populous city of Iran and the capital of Fars Province, located in the south of the country.

### Study population and sample size

The study population consisted of all households in Shiraz. The sample size was determined to be 740 households (2357 persons) through the use of the following formula and taking into account α = 0.05, d = 0.05, *p* = 0.6, q = 0.4, design effect = 2, and the confidence interval of 95%.

$$ \mathrm{n}=\frac{{\mathrm{Z}}_{1-\frac{\alpha }{2}\mathrm{pq}}^2}{{\mathrm{d}}^2}=370\kern0.5em 370\times 2=740 $$

### Sampling method and data collection procedure

In this research, a multi-stage sampling method was used. In the first stage, the city of Shiraz was divided into 11 municipal districts, each was considered as a stratum. The number of sample households in each stratum was determined using the stratified sampling method proportional to size and the number of households in that stratum. In the second stage, once the sample size in each stratum was determined, the urban neighborhoods within the strata were considered as clusters and the Excel 2007 software was used to randomly select a neighborhood from each district in order to collect the required data. In the third stage, the first home in the closest alley on the right side of the southwest side of each neighborhood selected in the second phase was considered as the first household to be studied. Then, based on the number of households in each neighborhood and the sample size determined for that district, and through using the systematic random sampling method, the households were selected and studied.

### Research/survey instrument

In order to collect the required data in this study, the WHO questionnaire entitled “World Health Survey”, which was developed in 2003 to measure the performance of health systems, was used. The questionnaire had been translated into Persian by Kavousi et al. (2012), and its validity and reliability had been already confirmed [22]. It consisted of seven main sections, including household socioeconomic data, household expenditures, average monthly income, demographic characteristics of each household member, the presence of a person in need of care in the household, the total costs of the household for outpatient services (over the last 1 month), and the total household costs for inpatient services (over the last 12 months). The data were collected from January 2018 to April 2018.

### Data analysis method

First, all the data collected from the households in Shiraz were entered into the Excel 2007 software and the households were classified into quintiles based on their income. The number of households in each quintile was then determined, and the healthcare costs of each quintile were calculated separately. In order to international comparison, the income and expenses were changed into the international dollars using the purchasing power parity (PPP) $ exchange rate of 42,000 Rials per 1 PPP$ in the study year, according to the World Bank website [23, 24].

To analyze the collected data and calculate the Gini coefficient, the concentration index and, consequently, the Kakwani index for the year under study, the Stata14.0 software was used and the related tables and diagrams were drawn.

According to the criticisms about the Fair Financial Contribution Index, such as the inability to distinguish between the regressive and progressive health financing systems as well as its inability to distinguish between horizontal and vertical equity, the Kakwani index, which is more acceptable, was used in the current study. This index can show the regressivity or progressivity of the studied health financing system, and is a valuable index in the measurement of equity in health financing [25].

In the present study, the Kakwani Progressivity Index (KPI) was calculated for each type of health service payments using the following formula [26]:

$$ \mathrm{KPI}=\mathrm{CI}-\mathrm{G} $$

Where CI=Concentration Index, and G = Gini coefficient.

The range of the Kakwani index is from-2 to + 1. If the index is greater than zero, there will be progressive financing, and if it is less than zero, the financing will be regressive [27].

The Gini coefficient (G) was determined using the following formula for identifying the income equity or inequity:

$$ \mathrm{G}=1\hbox{-} {\sum}_{i=0}^{k-1}\left({y}_{i+1}+{y}_i\right)\kern0.75em \left({x}_{i+1}-{x}_i\right) $$

Where x_i_ = cumulative proportion of population, and y_i_ = cumulative proportion of income

The income equity or inequity (the Gini coefficient) is derived from the Lorenz curve. This curve shows the cumulative proportion of the population from the poor to the rich, along with the cumulative proportion of income. The Gini coefficient is double the area between the Lorenz curve and the line at 45 degrees (indicating perfect equality of income). If the income is distributed equally among people, the curve will be aligned with the line at 45 degrees. The range of the Gini coefficient changes is between zero and one. If the Gini coefficient is zero, there will be perfect equality of income distribution. In contrast, if the Gini coefficient is one, there will be complete inequality in the distribution of income or expenditures [28].

The concentration curve shows the cumulative proportion of the population ranked by income, along with the cumulative proportion of healthcare payments. When the payments are distributed equally among the population, the payment concentration curve will be aligned with the line at 45 degrees (i.e. perfect equality of income). In this study, the concentration index was determined using the following formula to determine the equity or inequity in healthcare payments [27]:

$$ C=\left({p}_1{L}_2-{p}_2{L}_1\right)+\left({p}_2{L}_3-{p}_3{L}_2\right)+\dots +\left({p}_{T-1}{L}_T-{p}_T{L}_{T-1}\right) $$

Where L = cumulative proportion of payments, and  *P* = cumulative proportion of population.

The range of the concentration index (CI) changes is between − 1 and + 1. If the concentration index is + 1, all health expenditures have been paid by the richest person in the population. If the concentration index is − 1, all health expenditures have been paid by the poorest person in the population, and if it is equal to 0, the payments are proportional to income.

### Ethical statement

This study was approved by the Shiraz University of Medical Sciences Ethics Committee (Code: IR.SUMS.REC.1397.126). Oral informed consent was obtained from all participants in this study and all of them were assured of the confidentiality of their responses.

## Results

The results showed that most of the persons in the studied households were female (51.5%), married (57.6%), in the age group of 17–34 (35.8%), students (% 22.7), and had university degrees (45.6%), basic health insurance coverage (95%), especially Social Security Health Insurance (64.5%), and had no supplementary health insurance coverage (57.9%).

As Table 1 shows, the third quintile had the highest (91.10%) and the first quintile had the lowest (81.98%) numbers of referrals for receiving outpatient services. Also, the first quintile had the highest (55.41%) and the fifth one had the lowest (11.76%) visits to the public centers for receiving outpatient services. Regarding the use of private centers providing outpatient services and the use of both public and private services (combined services), the fifth quintile had the highest number of referrals (47.06 and 41.18%, respectively) and the first quintile had the lowest ones (17.52 and 27.07%, respectively).

**Table 1:**
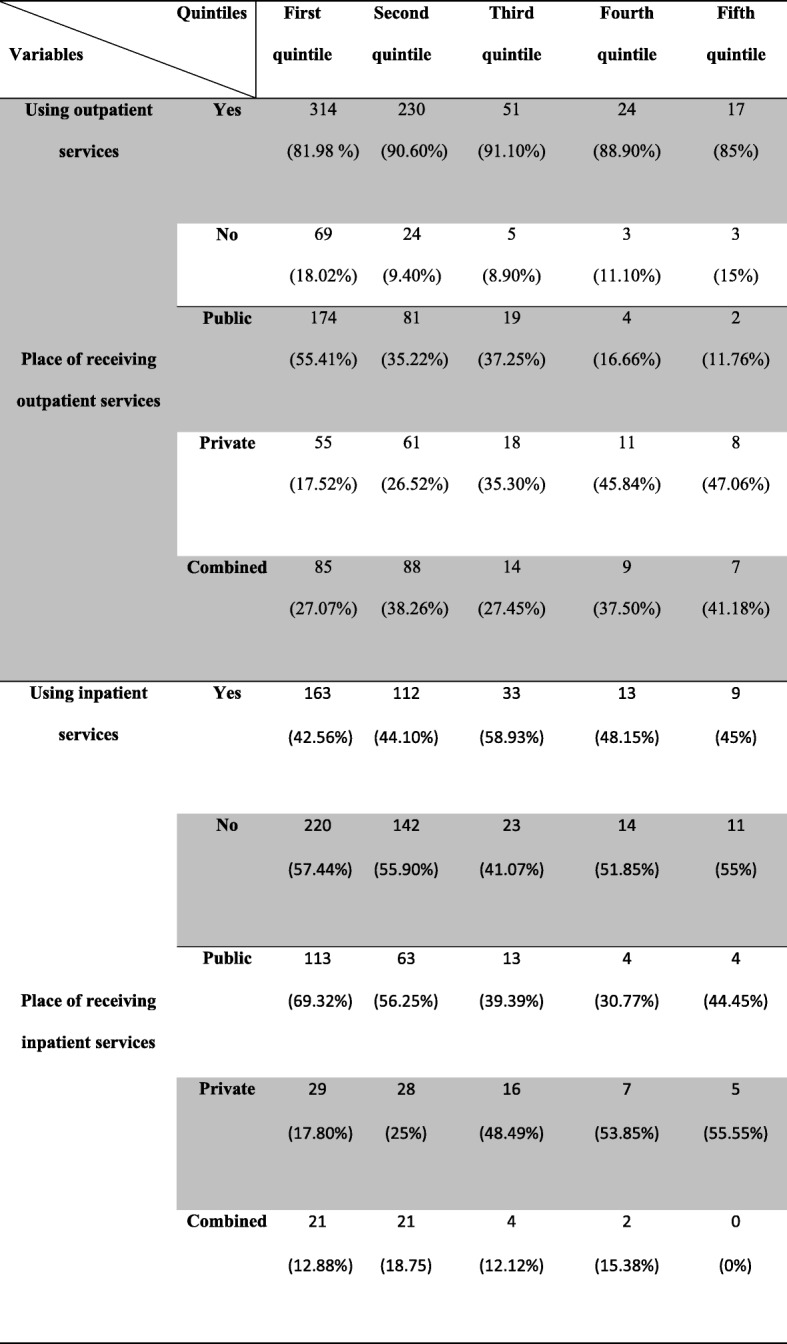
Referrals or non-referrals for and the place of receiving outpatient and inpatient services among the studied households in Shiraz, Iran

The third quintile had the highest (58.93%) and the first one had the lowest (42.56%) rates of using inpatient services. Besides, the first quintile had the highest (69.32%) and the fourth one had the lowest (30.77%) referrals to the public centers for receiving inpatient services. In the case of private centers providing inpatient services, the fifth quintile had the highest (55.55%) and the first one had the lowest (17.80%) referrals. Finally, the second quintile had the highest rate of using combined services (18.75%) and the fifth one, with the lack of using such services, had the lowest rate.

Moreover, as shown in Table 2, on average, the fifth and the first quintiles had the highest and the lowest annual income, respectively. Also, the findings of this table show that, on average, the highest and lowest health insurance premiums and outpatient costs were related to the fifth and the first quintiles, respectively, and the highest and lowest payments for inpatient services were related to the fourth and first quintiles, respectively. In addition, the fourth quintile had the highest and the first quintile had the lowest out-of-pocket payments (for costs related to both outpatient and inpatient services), and totally, the fifth and the first quintiles had paid the most and the least payments for health expenses, on average.

**Table 2:**
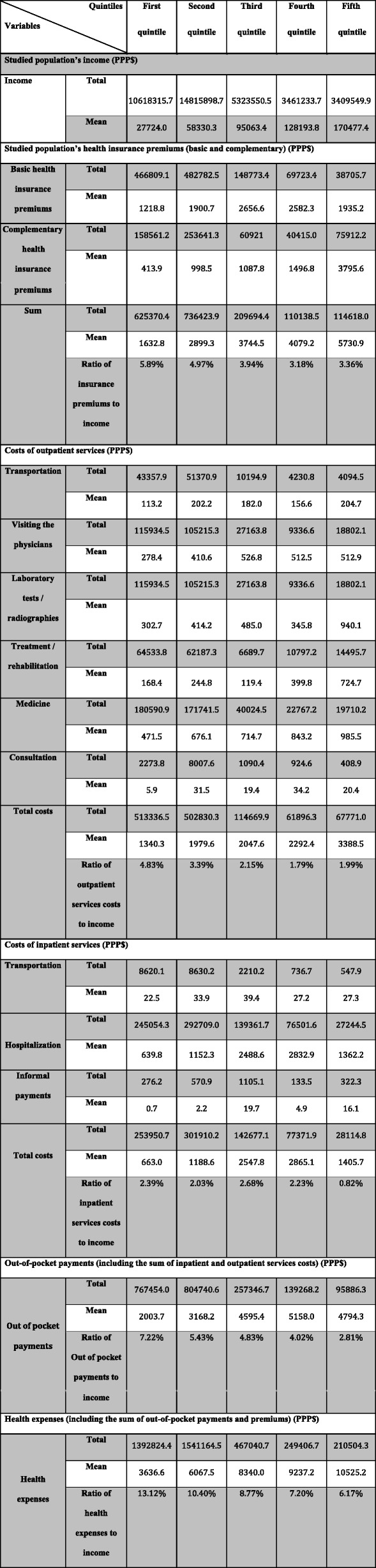
The studied househols’ income and the costs (PPP$) related to health insurance premiums, outpatient services, inpatient services, out-of-pocket payments, and health expenses among the studied households in Shiraz, Iran

However, it should be noted that the first and the fourth quintiles had respectively the highest and the lowest health insurance premiums and outpatient payments compared to their income. The third quintile had the highest and the fifth one had the lowest inpatient costs compared to their income. Finally, the first quintile had the most and the fifth one had the least out-of-pocket payments and health expenses compared to their income.

**Fig. 1:**
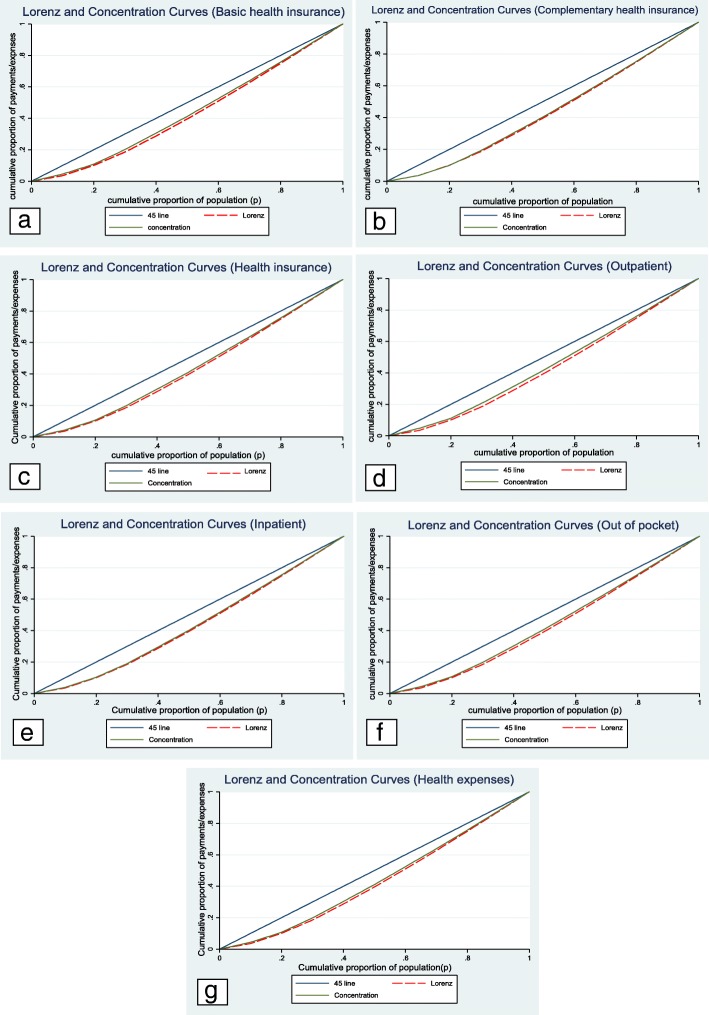
Concentration and Lorenz curves for all studied types of payments

According to Fig. 1a-g, in which the concentration curves for all types of payments have been located above the Lorenz curve as well as Table 3, the Kakwani index for all studied types of payments and, in total, for health expenses was negative, implying a regressive system for health costs.

**Table 3:**
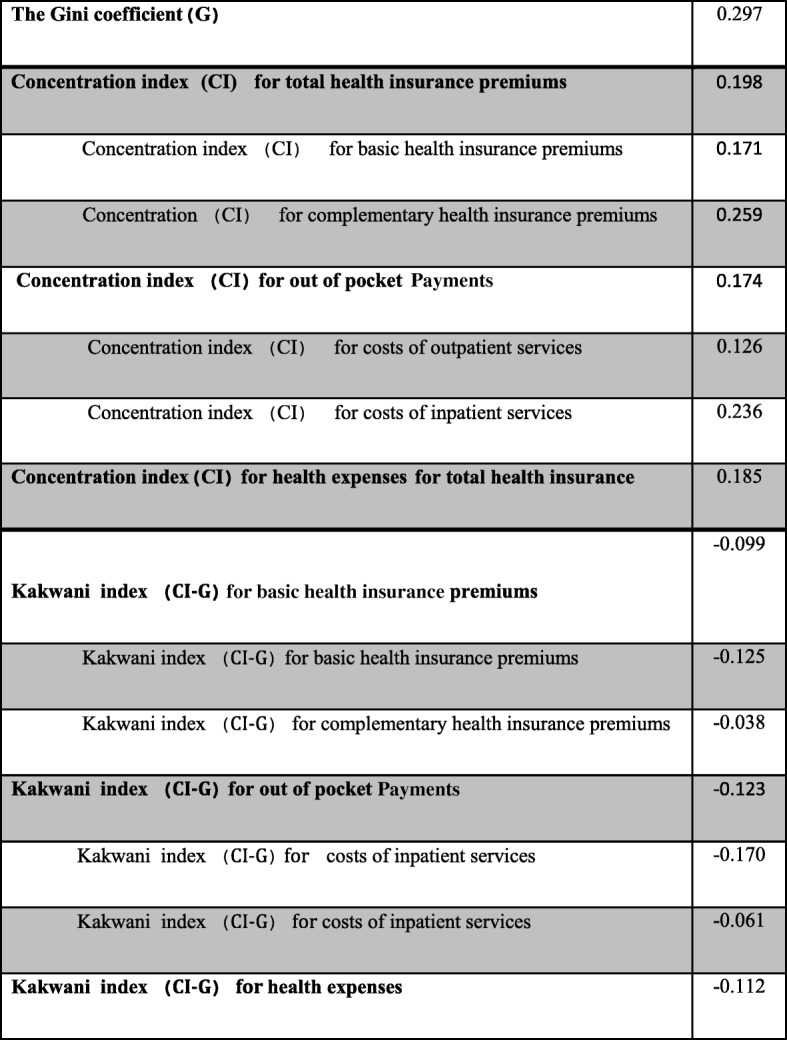
The Gini coefficient, Concentration index, and Kakwani index among the studied households in Shiraz, Iran

## Discussion

In recent years, the pressure of the growing people’s demand in different countries for better health and equity in health has become these issues a top priority in political affairs. Therefore, one of the goals of the health sector in each society is to prevent the impact of socioeconomic inequalities on the health of individuals and the associated costs [29]. This study was conducted with the aim of evaluating the level of equity in financing health expenses among households in Shiraz, Iran in 2018.

The results of this study showed that the Gini coefficient among the studied households in 2018 was 0.297, indicating a low level of income inequality and fairly equal distribution of wealth and income among population quintiles. The Gini coefficients in Iran had been 0.37 according to the Statistics Center of Iran (2015), while it was 0.362 [19], 0.387 [30], 0.424 [8], and 0.140 [31] in the studies conducted by Oliaeemanesh et al. (2018), Kazemian et al. (2017), Almasian Kia et al. (2015), and Hadian et al. (2014), respectively.

The concentration index of out-of-pocket payments (including the total costs of outpatient and inpatient services) in Shiraz districts was equal to 0.174. Given that the concentration index of out-of-pocket payments had a positive value, it could be concluded that the out-of-pocket payments were more concentrated on the rich households than on the poor ones. This might be due to the factors such as the lower use of costly health services by poor households, compared with rich ones, and more referrals of high-income families to the private health centers. The concentration index for out-of-pocket payments in Iran based on the findings of the studies carried out by Fazaeli et al. (2018), Kazemian et al. (2017), and Almasian Kia et al. (2015) had been 0.331, 0.056, and 0.256, respectively [8, 30, 32], which is consistent with that of the present study according to the Balsa et al.’ study [33]. However, in the study conducted by Rezapoor et al. (2016) on the urban population of Kerman, the concentration index for out-of-pocket payments was − 0.385 [34], which is not consistent with the results of the present study.

The concentration index of health insurance premiums for the households in Shiraz was the positive value of 0.198, which could indicate that high-income households paid more premiums. It can be due to that high-income households usually tend to use insurance packages that include more services, such as luxury health services, which are usually more expensive and, therefore, they pay higher premiums. The concentration index for the costs of outpatient and inpatient services in the present study was also positive and equaled to 0.126 and 0.236, respectively. One reason why the concentration index of healthcare expenses was positive could be the lack of demand for more expensive services by lower-income quintiles and their more frequent referrals to the public centers for receiving services due to their low financial power. In their study in Tehran, Ghafoori et al. (2014) obtained a concentration index of 0.105 for outpatient services, which is consistent with the results of the present study, and 0.015 for inpatient services, which is inconsistent with the results of the current study [35]. However, according to the results of the Rezapoor et al.’s study (2015), the concentration index for outpatient and inpatient services in the city of Kerman was, respectively, − 0.38 and − 0.435 [36], which are not similar to the results of the present study. In the present study, the concentration index of health expenses in the studied households had the positive value of 0.185, which could be indicative of the fact that on average, the high-income households (fifth quintile) of the studied population paid more for healthcare services, and the payment concentration was higher in this group. Raghfar et al. (2013) also reported that the concentration index of health expenses in Iran during the study years (1984–2009) was positive, implying that the health expenses were greater in higher deciles than lower ones. They argued that this could be due to the lower financial power of low-income deciles to pay the costs and their lack of access to health services [37]. The results of their study were consistent with those of the present one.

As previously stated, the Kakw ani index is affected by the distribution of income (Gini coefficient) and payments (concentration index). In the present study, the Kakwani index for out-of-pocket payments, basic health insurance premiums, complementary health insurance premiums, total health insurance premiums (basic and complementary), outpatients services costs, inpatient services costs, and health expenses was respectively − 0.123, − 0.125, − 0.038, − 0.099, − 0.171, − 0.061, and − 0.112, indicating that the health system burden was mainly on the low-income quintiles (first and second). According to Wagstaff et al. [38], the Kakwani index for the costs of outpatient services in the present study was relatively lesser extent regressive, but it was far lesser regressive for other studied payments and costs. The Kakwani index was more regressive for the costs related to the outpatient services than other costs and payments, possibly due to providing the most of these services by the private centers, because in case of the need for outpatient services, the people in the low-income quintiles had to go to the private centers and pay a larger share of their income to receive such services because of lower insurance coverage for these services. The results of Moradi’s study (2010) showed that the Kakwani index for out-of-pocket payments in Iran was − 0.022 [39], which is in line with the results of the current study. Hajizadeh et al. (2010) found that the Kakwani index of urban and rural areas in Iran was negative for out-of-pocket payments, and positive for social insurance premiums and health expenses, indicating that the payments were fairly distributed among different deciles [40]. Their results of the positive values of the Kakwani index are not in line with those of the present study. In addition, the Iranian Kakwani index for health expenses in the study carried out by Raghfar et al. (2013) during the studied years (1984–2009) was positive and, therefore, progressive [41]. This is not consistent with the results of the current study.

The similar foreign studies have had different results. For example, a study carried out to evaluate the progressivity of the social health insurance systems in the OECD countries showed that the Kakwani index in Germany and the Netherlands was negative, given that the people in high-income groups did not have to pay compulsory premiums and could withdraw the insurance schemes. But it was positive in France, because the people were not allowed to withdraw [38]. Another study in Tanzania showed that the Kakwani index for social health insurance premiums was 0.27 [42]. The mean Kakwani index for out-of-pocket payments was − 0.2 in a study conducted in Slovakia, because a large number of medications in this country were not covered by the health insurance system and the people had to buy the medicines directly through out-of-pocket payments [4]. The Kakwani index for out-of-pocket payments was also negative in the studies carried out in African countries, including Côte d’Ivoire, Guinea, Senegal and Tanzania [42, 43].

The differences between the results of the present study and those of other studies conducted in Iran can be due to the differences in the studied households’ socio-economic status and income level, the number of public and private service providers, and the illness behavior among different provinces, as well as the databases used, the year of study and the related inflation rate and therefore the percentage of household income spent on the health care costs, etc. Also, the differences between the results of the present study and those of other studies conducted in other countries mentioned above can be due to such reasons.

In general, the results of this study showed that the financial burden of health expenses was on the lower-income people (first and second quintiles). Fairness and distribution of the financial burden of out-of-pocket payments and health insurance premiums and, in general, healthcare expenses are very important. If the government is unable to finance health care services, the financing burden will be directly borne by the people, so that they will have to pay for their treatment costs and expenses as out-of-pocket payments. Although the out-of-pocket payment is common in developed and developing countries, it is the most inefficient way of financing the health system. In low-income countries, the lack of or poor health insurance coverage and inadequate social support have led households to frequently make out of pocket payments. Out-of-pocket payments can have serious negative impacts on the access to and use of services, especially by the poor, and this method of financing is often a regressive way of paying for health care services [17]. However, health financing policies based on the prepaid and public resources schemes, which have higher risk-pooling and better risk-sharing, can be a key strategy for achieving universal health coverage, reducing out-of-pocket payments, and ultimately, improving the quality of health care in poor and developing countries [44].

Among the limitations of this study are the dispersion of the study population and samples, the lack of cooperation of some heads of households with the researchers for various reasons, the lack of access to the tax data paid by the study population as a source of health financing, and the lack of weighing out-of-pocket payments and health insurance premiums in performing calculations.

## Conclusion

According to the findings of the present study, it could be concluded that the financing of health expenses in Shiraz, Iran was regressive in 2018 and there was vertical inequity. In this regard, making a commitment to implementing universal insurance policies, reviewing how to receive premiums based on the people’s ability to pay, redistributing income in the health sector to support low-income groups, strengthening the health insurance schemes, modifying the health insurance benefit packages in order to cover the essential medical services, developing pro-poor strategies as well as pushing the targeted subsidy plan towards further financial support of the health sector can be recommended.

Overall, it can be said that the health financing policies based on prepayment schemes and public financing can result in higher risk pooling and better risk sharing and can provide more access to universal health coverage, lower out-of-pocket payments and, finally, progressive health financing system and improved equity in healthcare financing.

## Abbreviations

PPP: Purchasing power parity
WHO: World Health Organization
